# Circulating Proprotein Convertase Subtilisin/Kexin Type 9 Levels and Cardiometabolic Risk Factors: A Population-Based Cohort Study

**DOI:** 10.3389/fcvm.2021.664583

**Published:** 2021-05-10

**Authors:** Jie Shi, Xiaoyong Li, Weiwei Zhang, Yixin Niu, Ning Lin, Hongmei Zhang, Guang Ning, Jiangao Fan, Li Qin, Qing Su, Zhen Yang

**Affiliations:** ^1^Department of Endocrinology, Xinhua Hospital, Shanghai Jiao Tong University School of Medicine, Shanghai, China; ^2^Department of Endocrine and Metabolic Diseases, Shanghai Clinical Center for Endocrine and Metabolic Diseases, Ruijin Hospital, Shanghai Institute of Endocrinology and Metabolism, Shanghai Jiao Tong University School of Medicine, Shanghai, China; ^3^Shanghai Key Laboratory of Children's Digestion and Nutrition, Department of Gastroenterology, Xinhua Hospital, Shanghai Jiao Tong University School of Medicine, Shanghai, China

**Keywords:** proprotein convertase subtilisin kexin type 9, dyslipidemia, hypertension, type 2 diabetes, metabolic syndrome, cardiometabolic risk factors

## Abstract

**Aims:** To evaluate the prospective association of circulating PCSK9 levels with the cardiometabolic risk profiles (high LDL-cholesterol, high triglycerides, low HDL-cholesterol, hypertension, type 2 diabetes, and metabolic syndrome).

**Methods:** A population-based prospective study was conducted among 7,104 Chinese individuals (age 56.2 ± 7.5 years; 32.0% men). Circulating PCSK9 levels were measured using ELISA.

**Results:** Circulating PCSK9 levels were higher in women than men (286.7 ± 90.1 vs. 276.1 ± 86.4 ng/ml, *p* < 0.001). And circulating PCSK9 was positively correlated with LDL-cholesterol, total cholesterol, and triglycerides both in men and women (all *p* < 0.001). The positive correlation between PCSK9 and waist circumference, fasting glucose, insulin resistance, systolic blood pressure, diastolic blood pressure and C-reactive protein (all *p* < 0.01) was observed in women only. According to Cox regression analysis, circulating PCSK9 was positively associated with incidence of high LDL-cholesterol both in men (HR 1.33, 95% CI 1.09–1.65, *p* < 0.001) and women (HR 1.36, 95% CI 1.12–1.69, *p* < 0.001). Moreover, PCSK9 was significantly associated with incident high triglycerides (HR 1.31, 95% CI 1.13–1.72, *p* < 0.001), hypertension (HR 1.28, 95% CI 1.08–1.53, *p* = 0.011), type 2 diabetes (HR 1.34, 95% CI 1.09–1.76, *p* = 0.005), and metabolic syndrome (HR 1.30, 95% CI 1.11–1.65, *p* = 0.009) per SD change in women only. No statistically significant association was observed between circulating PCSK9 and incidence of low HDL-cholesterol (*p* > 0.1).

**Conclusions:** Elevated circulating PCSK9 was significantly associated with cardiometabolic risk factors and independently contributed to the prediction of cardiometabolic risks in women.

## Introduction

Proprotein convertase subtilisin/kexin type 9 (PCSK9), the ninth member of the proprotein convertase family, is a key lipid metabolic regulator (1, 2). It is reported that PCSK9 is a serine protease produced primarily by the liver and the intestine, but only the liver releases it into circulation (3). This protease regulates cholesterol homeostasis by promoting degradation of low-density lipoprotein (LDL) receptor, the major route of clearance of circulating LDL-cholesterol, through an endosomal/lysosomal pathway (4). Subsequently, reduced LDL receptor levels resulted in impaired clearance of LDL-cholesterol, leading to hypercholesterolemia and metabolic disorders. Genetic studies have shown that gain-of-function mutations of the *PCSK9* gene caused hypercholesterolemia (5), whereas loss-of-function mutations in *PCSK9* resulted in hypocholesterolemia and reduced risk of cardiovascular disease (6, 7). On account of the involvement of PCSK9 in the degradation of LDL receptor, this crucial lipid metabolic regulator has recently emerged as new and promising pharmacological targets for the management of atherosclerotic cardiovascular disease. Indeed, treatment targeted at PCSK9 inhibition markedly reduced atherogenic lipoproteins and confers additional cardiovascular benefit beyond that achieved by lipid-lowering treatment alone (8–10).

Although the best-described impact of PCSK9 is on the levels of LDL-cholesterol, data from epidemiological studies have shown that PCSK9 is also associated with certain features of cardiometabolic profiles including high-density lipoprotein (HDL) cholesterol, triglycerides, blood pressure, and fasting glucose, as well as insulin resistance (11–13). However, to date, evidence from prospective study about the relationship between circulating PCSK9 and incidence of cardiometabolic risk factors is scarce.

To further evaluate whether PCSK9 contributes to the cluster of cardiometabolic risk factors involved in atherosclerotic cardiovascular diseases, we investigated prospectively the incidence of these metabolic abnormalities in relation to baseline PCSK9 levels in a large-scale Chinese population.

## Materials and Methods

### Study Population

**Figure 1:**
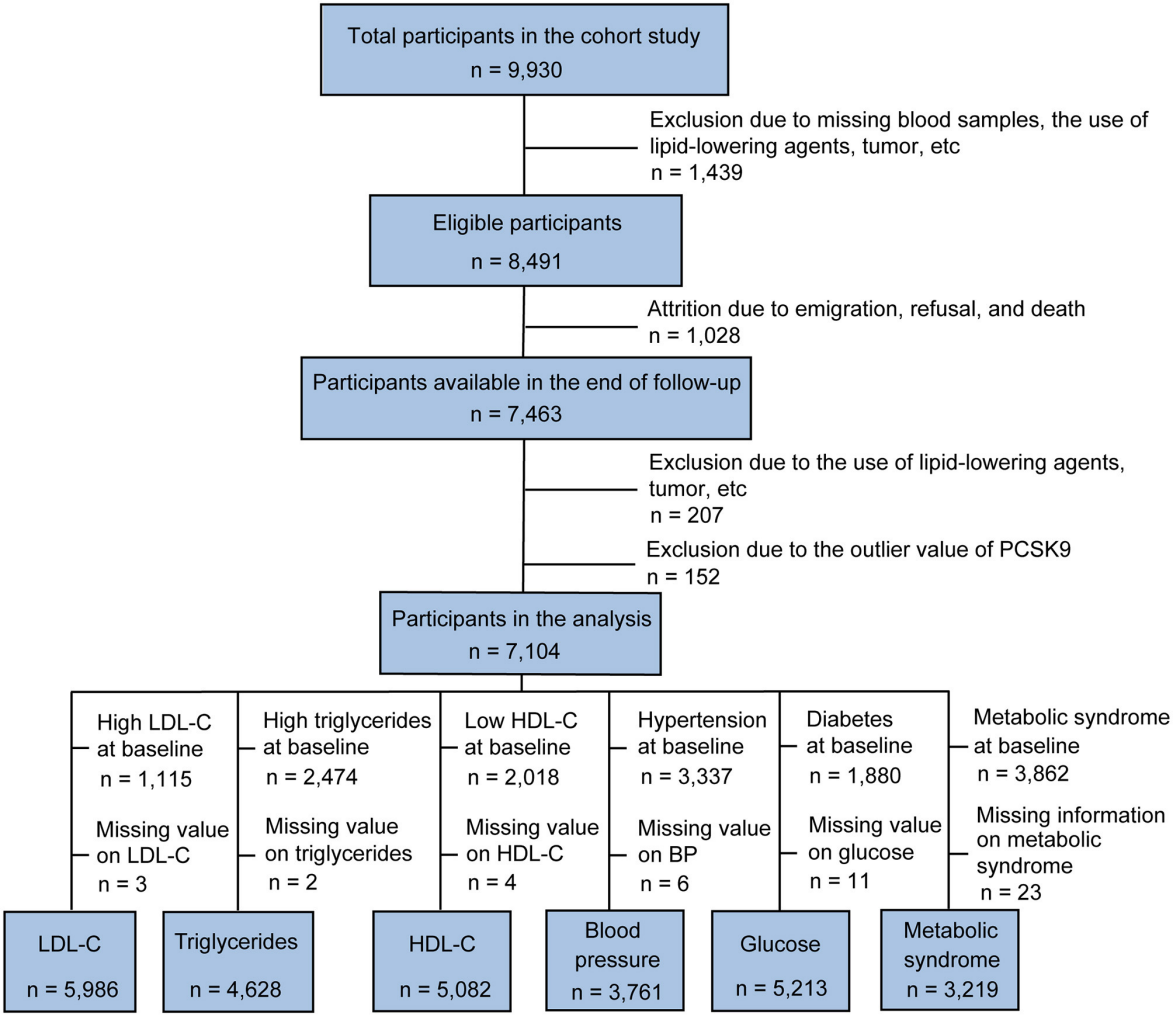
Overview of the study design.

Subjects were recruited from the China Cardiometabolic Disease and Cancer Cohort (4C) Study, a community-based study conducted among 259,657 Chinese individuals aged 40 years and older (14, 15). The 4C study was performed in 25 communities across mainland China. The study design and methods have been described previously in detail (14). The data presented in this article are based on the subsamples from the Chongming District, Shanghai, China. From May to November 2011, a total of 9,930 subjects (40–73 years of age) participated in the baseline survey. In 2014, participants returned for a 3.1-year follow-up investigation. Individuals with the following conditions were excluded from this study: missing blood samples, the use of lipid-lowering agents (such as statins, fibrates, and natural products), tumor, infectious or systematic inflammatory diseases, significant hematologic disorders, thyroid dysfunction, severe liver and/or renal insufficiency, and PCSK9 outliers, which were defined as the extreme value (lower or upper 1% of the distribution) or repeated measurements coefficient of variation >15%, resulting in the inclusion of 7,104 participants (2,273 men and 4,831 women) in the analysis (Figure 1).

The study protocol was approved by the Ethics Committee of Xinhua Hospital Affiliated to Shanghai Jiao Tong University School of Medicine. Written informed consent was obtained from all participants.

### Data Collection

A standardized questionnaire was used by trained physicians to collect essential information, including age, sex, lifestyle factors, educational attainment, physical activity, and previous medical history. Physical activity was evaluated based on the short form of the International Physical Activity Questionnaire by adding questions regarding the frequency and duration of moderate and vigorous activities and walking [Guidelines for data processing and analysis of the International Physical Activity Questionnaire (IPAQ)]. Current smokers or drinkers were defined as subjects who had a regular smoking or drinking status in the past 6 months. Anthropometric measurements, including height, weight, and waist circumference, were collected by certified medical staff using standard protocols. Blood pressure was measured with an automated electronic device (OMRON Model1 Plus; Omron Company, Kyoto, Japan).

### Laboratory Measurements

Venous blood samples were obtained after overnight fasting for at least 10 h and were collected in tubes containing EDTA. The blood samples were centrifuged at 4°C and stored at −80°C until analysis. Circulating LDL-cholesterol, HDL-cholesterol, total cholesterol, and triglycerides, were measured with an autoanalyzer (Hitachi 7080; Tokyo, Japan). Venous plasma glucose level was determined by the glucose oxidase method (ADVIA-1650 Chemistry System, Bayer, Leverkusen, Germany). Fasting insulin was measured by RIA (Linco Research, St. Charles, MO). The circulating C-reactive protein was determined by ELISA kit (DY1707, R&D Systems, Minneapolis, MN) as recommended by the manufacturer.

### PCSK9 Measurement

The circulating PCSK9 levels were measured in duplicate using a commercial ELISA kit (DY3888, R&D Systems, Minneapolis, MN) according to the manufacturer's instructions and compared with purified human PCSK9 standards.

### Definition of Cardiometabolic Risk Factors

A low HDL-cholesterol was defined as HDL-cholesterol <1.0 mmol/L in men or <1.3 mmol/L in women. A high LDL-cholesterol was defined as LDL-cholesterol ≥3.37 mmol/L. A high triglyceride was defined as triglyceride ≥1.7 mmol/L or treatment with a lipid-lowering medication. Hypertension was defined as systolic blood pressure ≥140 mmHg or diastolic blood pressure ≥90 mmHg or current use of antihypertensive treatment. Incident type 2 diabetes was diagnosed according to the American Diabetes Association 2010 criteria, which is defined as an FPG ≥ 7.0 mmol/L (≥126 mg/dL), 2-h PPG ≥ 11.1 mmol/L (≥ 200 mg/dL), or HbA_1c_ ≥ 6.5% (48 mmol/mol). The metabolic syndrome was defined based on the Joint Statement of the International Diabetes Federation Task Force on Epidemiology and Prevention; National Heart, Lung, and Blood Institute; American Heart Association; World Heart Federation; International Atherosclerosis Society; and International Association for the Study of Obesity (16).

### Statistical Analysis

The continuous variables with normal distribution are shown as means ± SD, and variables with skewed distribution are expressed as medians (interquartile range) and log-transformed to approximate normality before analysis. Categorical variables are reported as a percentage (%). The subjects were divided into two groups according to sex. For comparisons between groups, we performed an unpaired independent-samples Student *t*-test for normally distributed continuous variables and a non-parametric Mann-Whitney U test for skewed distributed variables. The Chi-squared tests were applied to compare categorical variables. The Pearson correlation analysis was used to evaluated correlation coefficients between baseline circulating PCSK9 levels and metabolic parameters. Multivariate Cox regression analysis was run to determine the potential association between baseline PCSK9 levels and incidence of cardiometabolic risk factors. We also used restricted cubic splines with five knots at the 5th, 35th, 50th, 65th, and 95th centiles to flexibly model exploring the association of circulating PCSK9 levels on a continuous scale with the incidence of cardiometabolic risk factors. Hazard ratios (HRs) and 95% confidence intervals (CIs) for the relationship between PCSK9 and the incidence of cardiometabolic risk factors were generated with the Cox proportional hazards model. In order to minimize the effect of potential confounding factors, covariates were selected based on biologic interest, well-established risk factors for cardiometabolic diseases, or associated exposures and outcomes. Variables showing *p* < 0.05 in the univariable regression model were entered into the multivariable model. Multivariable adjusted models were used to explore the independent effect of PCSK9 on cardiometabolic risk factors. Data management and statistical analyses were performed with SPSS software (version 25.0) and R version 3.6.1. The significance level was set at *p* < 0.05, and *p*-values were provided for two-sided tests.

## Results

### Baseline Characteristics of Subjects

Table 1 summarizes the baseline characteristics of 7,104 participants who were stratified according to sex. The mean age of the subjects was 56.2 ± 7.5 years, and 32.0% of the participants were males. Circulating PCSK9 levels were higher in women than men (286.7 ± 90.1 ng/mL vs. 276.1 ± 86.4 ng/mL, *p* < 0.001).

**Table 1:** Baseline characteristics of the study participants (*n* = 7,104).

	**Total (*n* = 7,104)**	**Men (*n* = 2,273)**	**Women (*n* = 4,831)**	***p-*value**
PCSK9, ng/mL	283.3 ± 88.7	276.1 ± 86.4	286.7 ± 90.1	<0.001
Age, years	56.2 ± 7.4	57.8 ± 7.1	55.5 ± 7.7	<0.001
Current smoker, *n* (%)	963 (13.6)	750 (33.0)	213 (4.4)	<0.001
Current drinker, *n* (%)	777 (10.9)	593 (26.1)	184 (3.8)	<0.001
Physical activity				0.69
Low	5,101 (71.8)	1,618 (71.2)	3,483 (72.1)	
Middle	1,517 (21.4)	493 (21.7)	1,024 (21.2)	
High	486 (6.8)	162 (7.1)	324 (6.7)	
Educational attainment				<0.001
0–6	1,589 (22.4)	434 (19.1)	1,155 (23.9)	
7–9	3,466 (48.8)	1,123 (49.4)	2,343 (48.5)	
≥10	2,049 (28.8)	716 (31.5)	1,333 (27.6)	
BMI, kg/m^2^	24.3 ± 4.7	24.4 ± 4.7	24.3 ± 4.7	0.31
WC, cm	83.4 ± 7.6	85.8 ± 7.8	82.3 ± 7.5	<0.001
SBP, mm Hg	131 ± 15	134 ± 16	129 ± 15	<0.001
DBP, mm Hg	81 ± 11	83 ± 11	80 ± 11	<0.001
FPG, mmol/L	6.3 ± 1.6	6.5 ± 1.7	6.2 ± 1.6	<0.001
PPG, mmol/L	8.8 ± 3.9	8.9 ± 4.1	8.8 ± 3.8	0.05
HOMA-IR	1.83 (1.30–2.66)	1.69 (1.16–2.52)	1.88 (1.37–2.72)	<0.001
CRP, mg/L	1.46 (0.76–2.65)	1.46 (0.78–2.70)	1.46 (0.76–2.63)	0.63
HDL-C, mmol/L	1.24 ± 0.33	1.18 ± 0.34	1.27 ± 0.32	<0.001
LDL-C, mmol/L	2.65 ± 0.76	2.58 ± 0.73	2.68 ± 0.78	<0.001
TC, mmol/L	4.70 ± 1.02	4.62 ± 0.97	4.74 ± 1.04	<0.001
Triglycerides, mmol/L	1.36 (0.96–1.98)	1.44 (0.99–2.24)	1.33 (0.95–1.89)	<0.001
Diabetes	1,880 (26.5%)	572 (25.2)	1,308 (27.1)	0.09
Metabolic syndrome	3,862 (54.4%)	1,219 (53.6)	2,643 (54.7)	0.39

*BMI, body mass index; CRP, C-reactive protein; DBP, diastolic blood pressure; FPG, fasting plasma glucose; HDL-C, high-density lipoprotein cholesterol; HOMA-IR, homeostasis model assessment of insulin resistance; LDL-C, low-density lipoprotein cholesterol; PCSK9, proprotein convertase subtilisin/kexin type 9; PPG, postprandial plasma glucose, SBP, systolic blood pressure, WC, waist circumference. Data are shown as means ± SD for normally distributed continuous variables, or median (interquartile range) for skewed distributed continuous variables*.

### Correlation of Baseline Circulating PCSK9 Levels With Clinical Characteristics

According to the Pearson correlation analysis, circulating PCSK9 levels were significantly and positively correlated with LDL-cholesterol, total cholesterol, and triglycerides (all *p* < 0.001) both in men and women (Table 2). Moreover, a positive correlation was observed between circulating PCSK9 and age, BMI, waist circumference, fasting plasma glucose, systolic and diastolic blood pressure, HOMA-IR, and C-reactive protein (all *p* < 0.01) in women only.

**Table 2:** Pearson correlation analysis between baseline circulating PCSK9 and clinical characteristics.

**Variables**	**PCSK9**					
	**Total**		**Men**		**Women**	
	***r***	***p-*value**	***r***	***p-*value**	***r***	***p-*value**
Age	0.080	<0.001	0.083	0.142	0.161	<0.001
BMI	0.069	<0.001	0.003	0.644	0.103	<0.001
WC	0.070	<0.001	0.029	0.235	0.124	<0.001
HDL-C	0.087	0.011	0.097	0.009	0.068	0.02
LDL-C	0.171	<0.001	0.164	<0.001	0.173	<0.001
Total cholesterol	0.249	<0.001	0.286	<0.001	0.231	<0.001
Log triglycerides	0.194	<0.001	0.183	<0.001	0.213	<0.001
FPG	0.067	<0.001	0.025	0.395	0.121	<0.001
PPG	0.047	0.052	0.032	0.271	0.093	<0.001
Log HOMA-IR	0.086	<0.001	0.006	0.765	0.153	<0.001
CRP	0.072	<0.001	0.068	0.107	0.112	<0.001
SBP	0.105	<0.001	0.065	0.083	0.132	<0.001
DBP	0.063	<0.001	0.026	0.207	0.092	<0.001

*BMI, body mass index; CRP, C-reactive protein; DBP, diastolic blood pressure; FPG, fasting plasma glucose; HDL-C, high-density lipoprotein cholesterol; HOMA-IR, homeostasis model assessment of insulin resistance; LDL-C, low-density lipoprotein cholesterol; PCSK9, proprotein convertase subtilisin/kexin type 9; PPG, postprandial plasma glucose, SBP, systolic blood pressure; WC, waist circumference*.

### Association Between Baseline Circulating PCSK9 Levels and Incidence of Cardiometabolic Risk Factors

**Figure 2:**
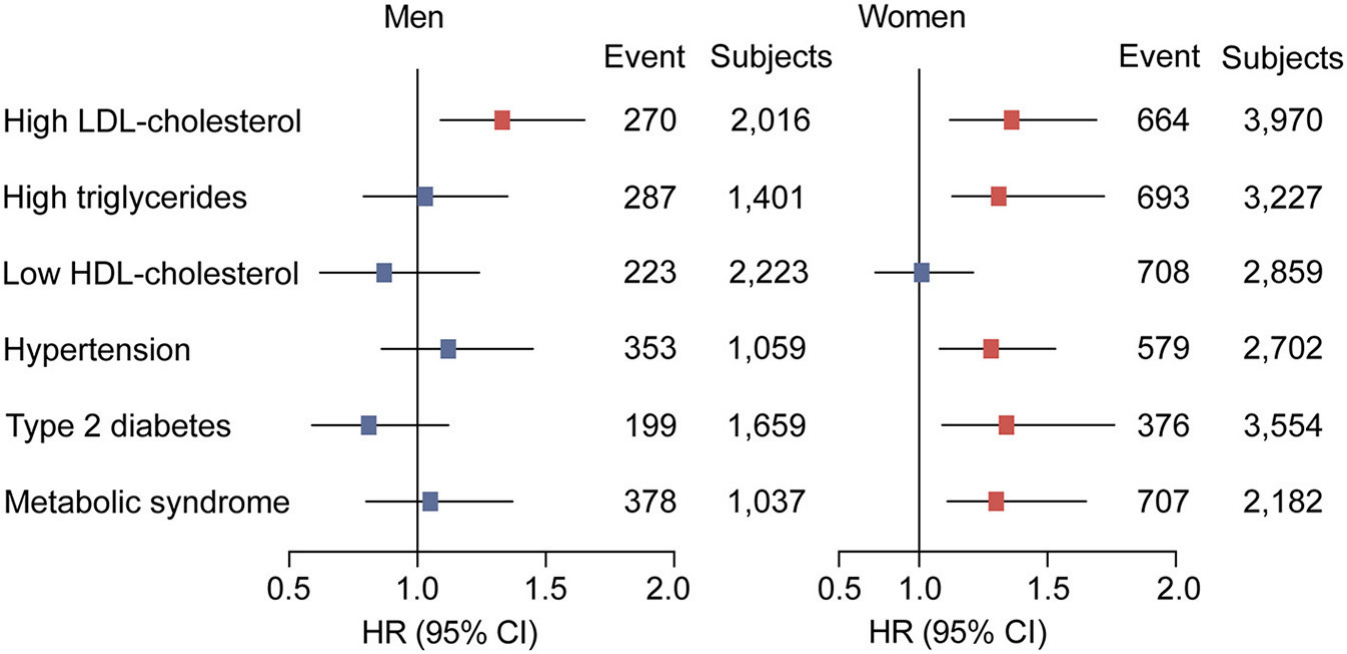
Adjusted hazard ratios (HRs) of cardiometabolic risk factors according to per 1-SD increment in baseline circulating PCSK9 levels in men and women. Model was adjusted for age, current smoking status, alcohol consumption, physical activity, educational attainment, BMI, waist circumference, C-reactive protein, fasting plasma glucose, post-loading plasma glucose, HOMA-IR, systolic blood pressure, diastolic blood pressure, and lipid profiles. Subgroup variable was excluded from the model.

**Figure 3:**
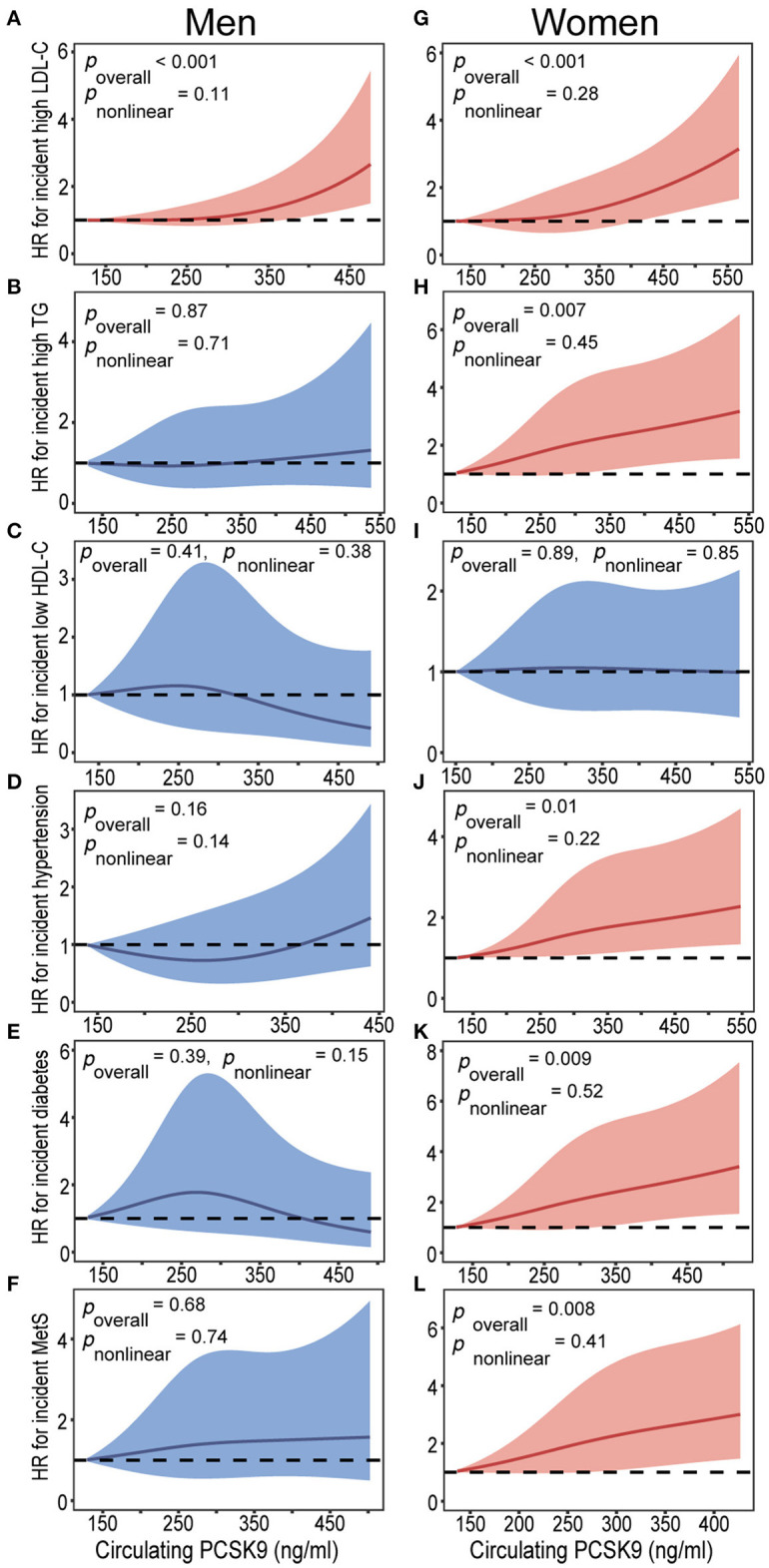
Baseline circulating PCSK9 levels on a continuous scale and incidence of cardiometabolic risk factors in men **(A-F)** and women **(G-L)**. Cardiometabolic risk factors including high LDL-C **(A,G)**, high triglycerides **(B,H)**, low HDL-C **(C,I)**, hypertension **(D,J)**, type 2 diabetes **(E,K)**, and metabolic syndrome **(F,L)**. Hazard ratios (HRs) are indicated by solid lines and 95% confidence intervals (CIs) by shaded areas. Model was adjusted for age, current smoking status, alcohol consumption, physical activity, educational attainment, BMI, waist circumference, C-reactive protein, fasting plasma glucose, post-loading plasma glucose, HOMA-IR, systolic blood pressure, diastolic blood pressure, and lipid profiles. Subgroup variable was excluded from the model. LDL-C, low-density lipoprotein cholesterol; TG, triglycerides; HDL-C, high-density lipoprotein cholesterol; MetS, metabolic syndrome; PCSK9, proprotein convertase subtilisin/kexin type 9.

Table 3 presents the hazard ratios (HR) for categorized cardiometabolic risk factors per 1-SD increment in baseline circulating PCSK9. Circulating PCSK9 was positively associated with incidence of high LDL-cholesterol both in men (HR 1.33, 95% CI 1.09–1.65, *p* < 0.001) and women (HR 1.36, 95% CI 1.12–1.69, *p* < 0.001) after adjustment for age, current smoking status, alcohol consumption, physical activity, educational attainment, BMI, waist circumference, C-reactive protein, fasting plasma glucose, post-loading plasma glucose, HOMA-IR, systolic blood pressure, diastolic blood pressure, and lipid profiles. Furthermore, a positive association was observed between circulating PCSK9 with incidence of high triglycerides (HR 1.31, 95% CI 1.13–1.72, *p* < 0.001), hypertension (HR 1.28, 95% CI 1.08–1.53, *p* = 0.011), type 2 diabetes (HR 1.34, 95% CI 1.09–1.76, *p* = 0.005), and metabolic syndrome (HR 1.30, 95% CI 1.11–1.65, *p* = 0.009) in women only (Figure 2). And the positive linear dose-response relationship was evident in the cubic spline regression model (Figure 3, *p* for non-linearity >0.1). No statistically significant association was observed between circulating PCSK9 and incidence of low HDL-C (*p* > 0.1) both in men and women.

**Table 3:** Multivariable Cox regression of cardiometabolic risk factors on per 1-SD increment in PCSK9.

	**Total**		**Men**		**Women**	
	**HR**	***p-*value**	**HR**	***p-*value**	**HR**	***p-*value**
	**(95% CI)**		**(95% CI)**		**(95% CI)**	
**High low-density lipoprotein cholesterol**						
Model 1	1.43 (1.19–1.72)	<0.001	1.31 (1.14–1.63)	<0.001	1.49 (1.23–1.79)	<0.001
Model 2	1.41 (1.18–1.70)	<0.001	1.32 (1.15–1.63)	<0.001	1.47 (1.22–1.76)	<0.001
Model 3	1.39 (1.17–1.68)	<0.001	1.29 (1.12–1.61)	<0.001	1.42 (1.20–1.71)	<0.001
Model 4	1.35 (1.12–1.68)	<0.001	1.33 (1.09–1.65)	<0.001	1.36 (1.12–1.69)	<0.001
**High triglycerides**						
Model 1	1.21 (1.07–1.37)	<0.001	1.03 (0.80–1.31)	0.84	1.28 (1.11–1.48)	<0.001
Model 2	1.20 (1.07–1.36)	<0.001	1.05 (0.82–1.34)	0.73	1.24 (1.08–1.44)	<0.001
Model 3	1.20 (1.06–1.35)	0.002	1.06 (0.82–1.37)	0.79	1.22 (1.06–1.42)	0.003
Model 4	1.19 (1.05–1.35)	0.006	1.03 (0.79–1.35)	0.66	1.31 (1.13–1.72)	<0.001
**Low high-density lipoprotein cholesterol**						
Model 1	1.05 (0.90–1.22)	0.55	0.85 (0.62–1.16)	0.30	1.07 (0.89–1.28)	0.37
Model 2	1.02 (0.87–1.18)	0.85	0.84 (0.61–1.15)	0.27	1.05 (0.87–1.25)	0.33
Model 3	0.99 (0.84–1.15)	0.87	0.81 (0.58–1.13)	0.25	0.98 (0.81–1.19)	0.46
Model 4	1.00 (0.85–1.19)	0.99	0.87 (0.62–1.24)	0.34	1.01 (0.83–1.21)	0.52
**Hypertension**						
Model 1	1.27 (1.11–1.45)	<0.001	1.12 (0.88–1.42)	0.35	1.36 (1.16–1.60)	<0.001
Model 2	1.25 (1.09–1.43)	<0.001	1.15 (0.90–1.46)	0.28	1.29 (1.09–1.52)	0.001
Model 3	1.24 (1.08–1.42)	0.002	1.17 (0.91–1.50)	0.23	1.26 (1.06–1.49)	0.006
Model 4	1.17 (1.05–1.36)	0.019	1.12 (0.86–1.45)	0.27	1.28 (1.08–1.53)	0.011
**Type 2 diabetes**						
Model 1	1.20 (1.11–1.39)	0.003	0.91 (0.68–1.21)	0.52	1.35 (1.13–1.61)	<0.001
Model 2	1.18 (1.10–1.37)	0.005	0.90 (0.67–1.20)	0.48	1.29 (1.10–1.51)	<0.001
Model 3	1.17 (1.09–1.35)	0.008	0.88 (0.65–1.18)	0.30	1.25 (1.10–1.49)	0.002
Model 4	1.14 (1.07–1.33)	0.020	0.81 (0.59–1.12)	0.21	1.34 (1.09–1.76)	0.005
**Metabolic syndrome**						
Model 1	1.27 (1.11–1.45)	<0.001	1.18 (0.93–1.49)	0.17	1.31 (1.11–1.67)	<0.001
Model 2	1.24 (1.08–1.43)	<0.001	1.19 (0.94–1.51)	0.19	1.27 (1.09–1.57)	0.004
Model 3	1.24 (1.07–1.42)	0.002	1.17 (0.92–1.48)	0.21	1.28 (1.09–1.59)	0.003
Model 4	1.22 (1.06–1.41)	0.007	1.05 (0.80–1.37)	0.32	1.30 (1.11–1.65)	0.009

*Model 1 was adjusted for age, current smoking status, alcohol consumption, physical activity, and educational attainment. Model 2 was further adjusted for BMI and waist circumference. Model 3 was further adjusted for HOMA-IR and C-reactive protein. Model 4 was further adjusted for fasting glucose, post-loading plasma glucose, systolic blood pressure, diastolic blood pressure, and lipid profiles. Subgroup variable was excluded from the model*.

## Discussion

In the present study, we found that subjects with higher circulating PCSK9 had progressively worse cardiometabolic risk profiles in women, including incident high LDL-cholesterol, elevated triglycerides, hypertension, type 2 diabetes, and metabolic syndrome. However, no significant relationship was observed between PCSK9 and cardiometabolic risk factors in males, except incidence of high LDL-cholesterol. To the best of our knowledge, this is the first prospective cohort study to investigate the association between PCSK9 and incidence of cardiometabolic risk factors.

The key and predominantly characterized activity of secreted PCSK9 is enhancing the degradation of the LDL receptor (17), a principal endocytic receptor that mediates the clearance and catabolism of LDL (18). As a consequence of reduced LDL clearance, circulating levels of LDL-cholesterol increasing, which is a well-established risk factor of cardiovascular disease. Moreover, multiple studies reported that PCSK9 is also involved in the degradation of very low-density lipoprotein receptor, apolipoprotein E receptor 2 (ApoER2), LDLR-related protein-1 (LRP1), and fatty acid transporter CD36 (19, 20). Additionally, the human evidence of the *PCSK9* (S127R) gain-of-function mutation genetic study revealed that PCSK9 dramatically increased the production rate of apoB (21). Then the effect on apoB resulted in an overproduction of very low-density lipoprotein, intermediate-density lipoprotein, and LDL. Therefore, the observed positive correlation between PCSK9 and intermediate-density lipoprotein, which is the triglyceride-rich LDL subfraction (22), raises the possibility of a significant contribution of intermediate-density lipoprotein to the positive relationship between PCSK9 and triglycerides. Furthermore, PCSK9 increases intestinal triglyceride-rich lipoprotein production and secretion through transcriptional and post-transcriptional mechanisms and then enhances triglycerides accumulation by targeting the intermediate-density lipoprotein receptor in the adipose tissue (23). No significant association was observed between PCSK9 and low HDL-cholesterol, thus, it is reasonable to speculate that PCSK9 may associate with atherosclerotic cardiovascular disease through a pathway not fully overlapping with reduced HDL-cholesterol.

The existence of a significant correlation between triglycerides and glucose metabolism has supported the investigation of a possible involvement of PCSK9 in glucose homeostasis and insulin resistance. In accordance with the previous findings (11, 13), in our female population circulating PCSK9 is positively associated with fasting glucose and insulin resistance, which involved in the initiation and progression of cardiometabolic disease. Previous study also found that high serum PCSK9 is associated with increased risk of new-onset diabetes after transplantation in renal transplant recipients (24). Notably, several studies showed that LDL-cholesterol-lowering *PCSK9* (rs11583680, rs11591147, rs2479409, and rs11206510) genetic variants were associated with higher circulating fasting glucose levels and increased risk of type 2 diabetes (25). However, other studies reported that the loss-of-function *PCSK9* (p.R46L) genetic variant was not associated with impaired glucose homeostasis in humans (26) and PCSK9 inhibition did not increase the risk of new-onset diabetes, nor did it worsen glycemia (27, 28). Further study is necessary to shed lights on the underlying mechanism of the association between PCSK9 and glucose homeostasis.

A positive correlation was observed between baseline circulating PCSK9 levels and blood pressure in females, which is in line with data obtained in other cross-sectional researches (11, 12). Genetic evidence from the Hypertension Genetic Epidemiology Network (HyperGEN) and the Reasons for Geographic And Racial Differences in Stroke study (REGARDS) studies have shown that *PCSK9* variation associated with blood pressure in African Americans (29). However, other investigation failed to identify a relationship between PCSK9 and blood pressure (30). Such an inconsistent association may be explained in part by a limited sample size and cross-sectional study design.

In line with the previous reports (11, 12, 24), we found PCSK9 was positively correlated with both BMI and waist circumference in females, suggesting that PCSK9 may be associated with obesity, especially visceral adiposity, the most prevalent features of the cardiovascular disease. Nevertheless, only a minor reduction of the cardiometabolic risk was yielded with per 1-SD increment in PCSK9 after further adjustment for BMI and waist circumference. Consequently, relationship between circulating PCSK9 and cardiometabolic risk factors in women may not be merely the link of excess adipose tissue.

The most striking finding in the present study was the positive relationship between circulating PCSK9 levels and incidence of high triglycerides, hypertension, type 2 diabetes, and metabolic syndrome only in female subjects. Consistent with the previous studies, correlations between PCSK9 and a variety of clinical characteristics, including age, BMI, LDL-cholesterol, triglycerides, glucose, blood pressure, and C-reactive protein, were either stronger or only present in females (11, 12, 31). Furthermore, evidence from small-scale research (aged 15–26 years) found that obesity and type 2 diabetes were associated with significantly higher levels of PCSK9 in America young women, but not in young men (31). In humans, many studies described circulating PCSK9 levels as being significantly higher in women than men, but differences have also been found in postmenopausal compared to premenopausal women as well as in pregnant compared to non-pregnant women. Therefore, it is reasonable to speculate a possible role for sex hormones in PCSK9 synthesis and/or metabolism. According to evidence from animal and human studies, PCSK9 is regulated by hormones such as estrogen, glucagon, insulin, growth hormone, and thyroid hormone (32). Previous studies reported that circulating PCSK9 concentration are decreased when endogenous estrogens are high in women (33), and pharmacologically elevated estrogen levels have been shown to lower PCSK9 levels both in animals and humans (34, 35). Conversely, several studies indicated that the difference in circulating PCSK9 levels between postmenopausal and premenopausal women appears to be independent of estrogen status (11), and estrogen at physiological concentrations does not affect hepatocyte PCSK9 expression in human (36). Overall, the clear molecular mechanisms underlying the sex differences remain unclear. More intensive investigations are needed to clarify the effect of gender on the association between PCSK9 and a variety of cardiometabolic abnormalities.

There are several limitations of this study. First, the use of a single baseline PCSK9 measurement to predict outcomes was a simplified and practical approach but was unable to assess the relationship between changes in PCSK9 levels and the incidence of metabolic disorders. Second, we did not record the type of diabetes among incident cases, but given that all the participants in our study were at least 40 years old at enrollment, these participants were unlikely to have type 1 diabetes because the fasting glucose was only mildly elevated and insulin levels were high normal. Third, due to the observational nature of the study, we cannot draw the causality from our findings. Thus, further studies are necessary to clarify the underlying mechanisms behind the association of PCSK9 with cardiometabolic dysregulation and to determine whether high PCSK9 level is a cause or a consequence of cardiometabolic abnormalities, or both. Forth, it is not clear whether our findings in middle-aged and elderly Chinese female individuals can be generalized to younger populations or individuals of other ethnicities. In our study, men and women are significantly different for most evaluated parameters. Therefore, we failed to assess its potential impact on differences in results between the two groups. Although we have adjusted for almost all potential confounding factors in the regression models. Further research should be undertaken among diverse groups and to shed lights on the effects of sex on associations of PCSK9 with cardiometabolic risk factors. Fifth, loss or gain of function of *PCSK9* gene mutations may have had potential impacts on the statistical results. However, due to the study design, we failed to analyze the *PCSK9* mutations status in present study.

In summary, our results indicate that subjects with higher circulating PCSK9 had progressively worse cardiometabolic risk profiles in women. These findings highlight the importance of elevated circulating PCSK9 levels in the development of atherosclerotic cardiovascular disease in female individuals. Moreover, based on our study, elevated circulating PCSK9 levels may emerge as novel target of efficacious prevention of cardiovascular disease in women.

## Data Availability Statement

The raw data supporting the conclusions of this article will be made available by the authors, without undue reservation.

## Ethics Statement

The studies involving human participants were reviewed and approved by the Ethics Committee of Xinhua Hospital Affiliated to Shanghai Jiao Tong University School of Medicine. The patients/participants provided their written informed consent to participate in this study.

## Author Contributions

JS, XL, and WZ drafted the manuscript and performed the experiments. ZY, QS, LQ, and GN conceived and designed the study. WZ, YN, JF, HZ, and NL recruited the subjects, processed samples, and contributed to the acquisition of data. JS, XL, WZ, YN, and ZY analyzed the data. ZY revised the manuscript. All authors contributed to the article and approved the submitted version.

## Conflict of Interest

The authors declare that the research was conducted in the absence of any commercial or financial relationships that could be construed as a potential conflict of interest.

## Abbreviations

BMI: body mass index
CI: confidence interval
DBP: diastolic blood pressure
FPG: fasting plasma glucose
HbA_1c_: glycated hemoglobin
HDL: high-density lipoprotein
HOMA-IR: homeostasis model assessment of insulin resistance
HR: hazard ratio
LDL: low-density lipoprotein
PCSK9: proprotein convertase subtilisin/kexin type 9
PPG: postprandial plasma glucose
SBP: systolic blood pressure
SD: standard deviation
TG: triglycerides.
